# Efficacy evaluation and predictive value of IL-20 and Apelin-13 after cataract surgery by phacoemulsification combined with IOL implantation

**DOI:** 10.3389/fmed.2025.1737194

**Published:** 2026-01-13

**Authors:** Shuang Liu, Min Yang, Shengwei Wu, Dawei Zhang

**Affiliations:** Department of Ophthalmology, Beijing Luhe Affiliated Hospital of Capital Medical University, Beijing, China

**Keywords:** Apelin-13, biomarker, cataract, efficacy prediction, IL-20, intraocular lens implantation, phacoemulsification

## Abstract

**Background:**

Phacoemulsification combined with intraocular lens (IOL) implantation is the standard treatment for cataract. A proportion of patients still experience suboptimal visual outcomes or postoperative complications. This study aimed to investigate the roles of Interleukin-20 (IL-20) and Apelin-13 as biomarkers for assessing visual outcomes and predicting postoperative prognosis following phacoemulsification with IOL implantation.

**Methods:**

In this retrospective study, 193 cataract patients who underwent phacoemulsification combined with IOL implantation between January 2023 and December 2024 were included. Based on the best-corrected visual acuity (BCVA) and complication status at 3 months postoperatively, patients were categorized into good (*n* = 138) and poor (*n* = 55) outcome groups. Clinical baseline data, levels of IL-20, Apelin-13 in preoperative serum and intraoperative aqueous humor were compared between two groups. Univariate and multivariate logistic regression analyses were employed to identify factors influencing postoperative outcomes. Receiver operating characteristic (ROC) curve analysis was used to evaluate the predictive performance of IL-20 and Apelin-13.

**Results:**

There were significant differences in diabetes history, CRP, and IL-6 levels (*p* < 0.05). Patients in the poor outcome group exhibited significantly higher IL-20 and lower Apelin-13 levels in both aqueous humor and serum (*p* < 0.05). Multivariate logistic regression analysis confirmed higher levels of IL-20 and lower levels of Apelin-13 in aqueous humor and serum were independent predictors of poor postoperative outcomes. ROC curve analysis indicated that levels of IL-20 and Apelin-13, as well as the IL-20/Apelin-13 ratio in aqueous humor effectively predicted postoperative outcomes.

**Conclusion:**

IL-20 and Apelin-13 are significantly associated with the efficacy of cataract surgery. Apelin-13 serves as a protective factor for favorable outcomes, whereas IL-20 is a risk factor. Their ratio shows strong predictive value for adverse prognosis. These biomarkers offer potential for preoperative risk assessment and personalized treatment strategies, with significant clinical implications for improving postoperative outcomes.

## 1 Introduction

Cataract is one of the major causes of blindness worldwide. According to the data from the World Health Organization, the incidence rate of cataract increases significantly with age, imposing a substantial burden on the elderly population. Among individuals aged over 60 years, the prevalence of cataract increases exponentially (1, 2). According to the Global Burden of Disease Study, disability adjusted life years (DALYs) caused by age-related cataracts have increased by 58% since 1990, with low-income countries bearing higher disease burdens due to controllable risk factors such as air pollution and metabolic diseases (3). Phacoemulsification combined with intraocular lens (IOL) implantation is the gold standard treatment for cataract, offering benefits including minimal tissue trauma, rapid postoperative recovery, and reliable visual outcomes (4). Phacoemulsification combined with IOL implantation is one of the most frequently performed surgical procedures globally, significantly improving vision and quality of life for millions of patients. Despite continuous advancements in surgical techniques and equipment leading to markedly increased success rates, postoperative outcomes vary individually among cataract patients. Some patients may still experience poor visual recovery or various complications, such as posterior capsular opacification, postoperative inflammatory response, and macular edema, all of which can affect visual quality and quality of life (5–7). Therefore, identifying novel biomarkers to predict postoperative outcomes and monitor visual recovery in cataract patients is of considerable clinical importance for the early identification of high-risk patients and guiding individualized treatment plans.

In recent years, inflammatory factors and peptides have been increasingly recognized for their key roles in the pathogenesis, progression, and prognosis assessment of various diseases. Cataract surgery by phacoemulsification combined with IOL implantation is an invasive procedure, and surgical trauma can induce intraocular inflammation. Following surgery, levels of various inflammatory mediators in the aqueous humor are changed, and multiple cytokines participate in the processes of postoperative tissue repair and complication development. The postoperative inflammatory response is thus a critical determinant of visual outcomes and complications risk (8, 9). The cytokine Interleukin-20 (IL-20) is a pleiotropic inflammatory factor, a member of the IL-10 cytokine family, and plays important roles in regulating inflammatory responses, angiogenesis, and tissue repair (10). Recent studies have found aberrant expression of IL-20 in ocular diseases such as diabetic retinopathy and uveitis, suggesting its potential involvement in blood-retinal barrier disruption and retinal inflammation. A recent study found a positive correlation between serum IL-20 levels and the occurrence of dry eye syndrome after cataract surgery, potentially mediated by IL-20 driven inflammatory cell infiltration and suppression of lacrimal gland function (11). However, the value of IL-20 in comprehensively assessing visual outcomes and predicting postoperative prognosis following phacoemulsification with IOL implantation remains unclear.

Apelin-13, an active peptide of the Apelin/APJ system, has been demonstrated to regulate angiogenesis, inflammation, and apoptosis, and shows potential protective effects in cardiovascular diseases, renal injury, and neurological disorders (12–16). Recent evidence indicates that Apelin-13 exerts neuroprotective effects in a mouse model of glaucoma, counteracting optic nerve injury by modulating glucose metabolism and alleviating oxidative stress (17). Similarly, in models of traumatic brain injury, Apelin-13 has been shown to confer neuroprotection via suppression of inflammatory and oxidative pathways (18).

Currently, direct research on IL-20 and Apelin-13 in the context of cataract and postoperative recovery remains limited. The predictive value of IL-20 and Apelin-13 for outcomes following phacoemulsification with IOL implantation has not been studied. However, given their recognized roles in inflammation, tissue repair, and vascular regulation, we hypothesize that IL-20 and Apelin-13 may hold potential for assessing and predicting outcomes following phacoemulsification with IOL implantation. Serum and aqueous humor, as reflections of the intraocular microenvironment (19, 20), may provide early clues through their levels of IL-20 and Apelin-13 for predicting postoperative efficacy in cataract patients. Therefore, this retrospective study analyzed the clinical medical records of cataract patients to explore the association between serum and aqueous humor levels of IL-20 and Apelin-13 and postoperative outcomes, analyzing their role in evaluating and predicting efficacy after phacoemulsification combined with IOL implantation, aiming to provide references for clinical individualized treatment and optimizing surgical plans for cataract patients.

## 2 Materials and methods

### 2.1 Study **population**

**Figure 1:**
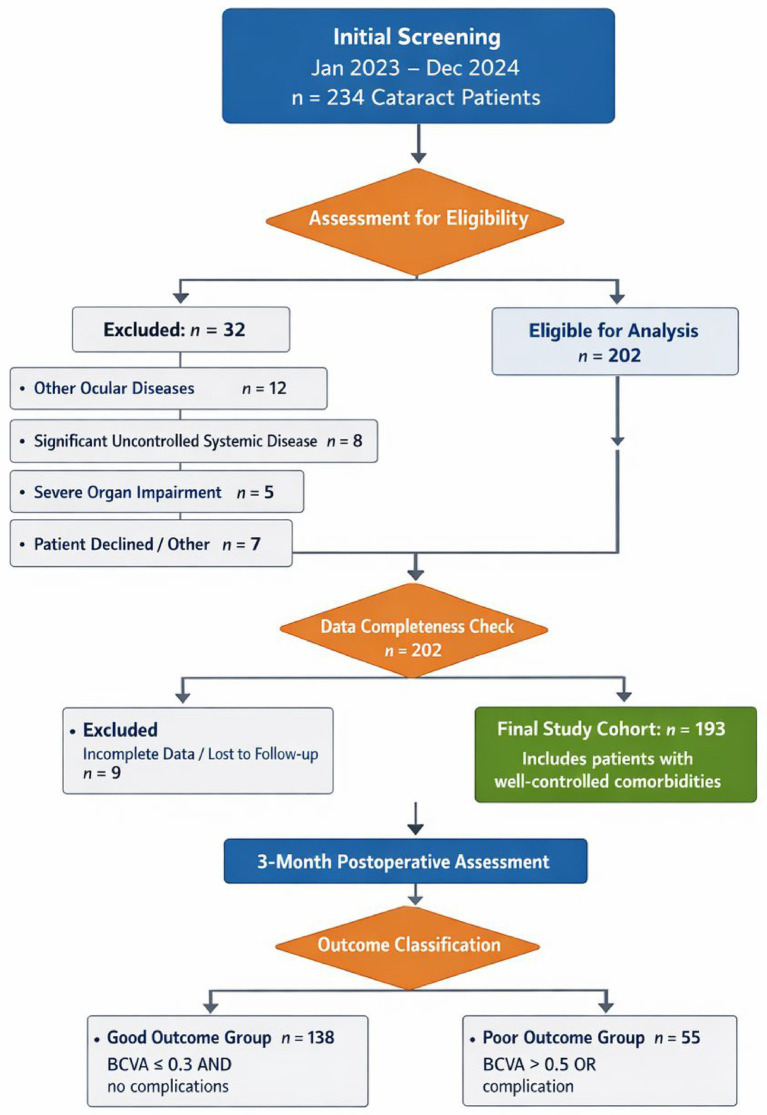
Flow chart of subject enrollment.

This retrospective study analyzed the medical records of 234 cataract patients who underwent phacoemulsification combined with IOL implantation at Ophthalmology department, Beijing Luhe Affiliated Hospital Capital Medical University hospital between January 2023 and December 2024. Among them, 32 patients were excluded based on the inclusion and exclusion criteria, and 9 were excluded due to incomplete clinical medical records or loss of follow-up. Ultimately, 193 patients were included as study subjects (Figure 1). Their clinical characteristics and laboratory indicator data were retrieved and collected from the hospital’s electronic medical record database.

Inclusion Criteria: (1) Patients clinically diagnosed with cataract; (2) Patients undergoing phacoemulsification combined with IOL implantation for the first time; (3) Patients voluntarily receiving treatment; (4) Absence of relevant infection or acute/chronic inflammatory reactions before treatment; (5) Complete clinical pathological data and follow-up data.

Exclusion Criteria: (1) Combined with other ocular diseases, such as diabetic retinopathy, uveitis, or history of intraocular inflammation; (2) Significant, uncontrolled systemic diseases (e.g., recent myocardial infarction, unstable angina, severe chronic obstructive pulmonary disease) that posed a high perioperative risk or were associated with profound systemic inflammation. Patients with well-controlled hypertension or hyperlipidemia on stable medication regimens were not excluded. (3) Patients with severe impairment of vital organs such as heart, liver, or kidney; (4) Patients with a history of ocular surgery or trauma; (5) History of drug allergy; (6) Patients with tumors; (7) Interruption of treatment or follow-up during the study period; (8) Incomplete clinical medical records and follow-up data.

Diagnostic Criteria: Decreased visual acuity and snowflake-like opacities in the lens region, along with classification as Grade III cortical cataract according to the Lens Opacities Classification System III (LOCS III). Other ocular diseases causing visual impairment were excluded. Routine examinations included uncorrected visual acuity, corrected visual acuity, intraocular pressure measurement, and slit-lamp examination. The LOCS III grading system was used to detail nuclear hardness, extent of cortical opacification, and characteristics of posterior subcapsular opacities. A red reflex test was recommended for each patient, with corneal endothelial cell count and ocular B-scan performed when necessary.

Patient Grouping: Based on the best-corrected visual acuity (BCVA, LogMAR visual acuity) and complication status at 3 months postoperatively, the 193 patients were divided into two groups: (1) Good outcome group: BCVA (LogMAR) ≤ 0.3, and absence of complications such as macular edema, persistent inflammation, corneal edema; (2) Poor outcome group: BCVA (LogMAR) > 0.3 and/or occurrence of postoperative complications requiring treatment.

### 2.2 Collection of patient medical record data

Patient case data were collected through the electronic medical record system, including: (1) General information such as age, gender, body mass index (BMI), course of disease, smoking history, alcohol history, comorbid hypertension, and comorbid hyperlipidemia. (2) Preoperative examination data such as BCVA, intraocular pressure, preoperative central macular thickness (CMT), and axial length. (3) Blood biomarkers, including levels of IL-20, Apelin-13, Interleukin-6 (IL-6), Procalcitonin (PCT), and C-reactive protein (CRP) were selected as they are canonical and clinically routine markers of systemic inflammation, providing a baseline against which to compare the novel ocular-specific biomarkers. (4) Collection of intraoperative aqueous humor samples for measuring IL-20 and Apelin-13 levels. (5) Intraoperative parameters, such as phacoemulsification time and phacoemulsification energy. (6) Postoperative follow-up data: BCVA, intraocular pressure, and slit-lamp examination were reviewed at 1 week, 1 month, 3 months, and 6 months postoperatively. Postoperative complications such as corneal edema, anterior chamber inflammation, posterior capsular opacification, macular edema, etc., were recorded.

### 2.3 Sample collection and testing

Preoperative Serum Collection: On the morning before cataract surgery, 3–4 mL of fasting venous blood was collected from all study participants. Whole blood samples were centrifuged at 4 °C, 3000 rpm for 10 min using a low-temperature ultracentrifuge to separate and collect serum, which was stored at −80 °C pending testing.

Aqueous Humor Collection: During phacoemulsification surgery, aqueous humor samples were collected from the operative eye of all subjects. After topical anesthesia, routine surgical disinfection, and draping, an anterior chamber paracentesis was performed using a 25-gauge needle attached to a syringe, inserted 1 mm inside the corneal limbus. Approximately 0.15–0.2 mL of aqueous humor was aspirated and immediately transferred to a 0.5 mL Eppendorf tube. The samples were then stored in an ultra-low temperature freezer at −80 °C for future analysis.

All serum and aqueous humor samples were processed and analyzed by a single, dedicated research technician at the Clinical Laboratory of Beijing Luhe Affiliated Hospital. The human IL-20 ELISA kit (R&D Systems, USA; Catalog # DY724) from lot # 2365891 (expiry: 03/2025) and the human Apelin-13 ELISA kit (Phoenix Pharmaceuticals, USA; Catalog # EK-057-23) from lot # PHX1022 (expiry: 02/2025) were used. All samples were analyzed in a single batch between January 15, 2024 and December 2, 2024 after a median storage duration of 8.5 months (range: 1–24 months) at −80 °C.

Assay performance was validated using internal duplicate samples and repeated control runs. The intra-assay coefficients of variation (CV) were 4.8% for IL-20 and 6.2% for Apelin-13 (based on *n* = 20 replicate samples across the assay range). The inter-assay CVs were 7.5% for IL-20 and 9.1% for Apelin-13 (based on *n* = 5 independent runs of control samples). The lower limit of detection (LLOD) was 1.0 pg./mL for IL-20 and 5.0 pg./mL for Apelin-13, as per the manufacturer’s specifications. The absorbance values for all kit-provided positive and negative controls fell within the certified ranges (IL-20 positive: 0.85–1.25 OD, negative: 0.05–0.15 OD; Apelin-13 positive: 0.70–1.10 OD, negative: 0.04–0.12 OD), confirming assay validity. No out-of-range control values were observed.

### 2.4 Surgical protocol

All surgeries were performed by the same experienced senior ophthalmologist, involving standard clear corneal incision phacoemulsification and implantation of foldable hydrophobic acrylic IOL. The surgical steps were as follows: Local anesthesia was applied to the eyeball. An incision was made at the superior corneal limbus; the conjunctiva was cut and dissected posteriorly to fully expose the sclera. After achieving hemostasis, a scleral tunnel incision was made 2 mm posterior to the corneoscleral limbus, parallel to the limbus, with a depth of half the scleral thickness. An appropriately sized incision was made according to the patient’s specific lens condition, followed by lamellar dissection diagonally toward the corneal limbus, entering the clear cornea to establish the scleral tunnel. After entering the anterior chamber, a continuous curvilinear capsulorhexis (CCC) approximately 6 mm in diameter was performed. Hydrodissection and hydrodelineation of the lens were performed. The incision was enlarged, and the phacoemulsification tip was inserted. Parameters were set to power 30–40%, vacuum 300–400 mmHg, and flow rate 25–30 mL/min. The lens cortex and nucleus were emulsified and aspirated. Residual lens cortex was cleared. A viscoelastic agent was then injected, the IOL was implanted into the capsular bag, its position was adjusted, the viscoelastic was removed, miosis was induced, and the incision was sealed.

### 2.5 Postoperative efficacy assessment indicators

Follow-ups were conducted at 1 week, 1 month, 3 months, and 6 months postoperatively, assessing the following indicators: (1) Primary efficacy indicator: Recovery of best-corrected visual acuity (BCVA) (converted according to the LogMAR chart). (2) Secondary efficacy indicators: Incidence and types of postoperative complications, such as time of onset and severity of posterior capsular opacification, incidence of macular edema, and degree of postoperative inflammatory response.

### 2.6 Statistical analysis

Statistical analysis and graphing were performed using SPSS 27.0 statistical software (SPSS Inc., Chicago, IL, USA) and R software. The Kolmogorov–Smirnov test was used to assess the normality of data distribution. Measurement data conforming to a normal distribution are expressed as mean ± standard deviation (*x* ± *s*) and were compared between groups using the independent samples t-test. Non-normally distributed measurement data are expressed as median (P25, P75) and were compared between groups using the Mann–Whitney U test. Categorical data are expressed as number of cases and percentage and were compared between groups using the Chi-square test.

Logistic regression analysis was used to calculate odds ratios (OR) and 95% confidence intervals (CI) to identify independent risk factors affecting the efficacy of cataract surgery by phacoemulsification combined with IOL implantation. Receiver operating characteristic (ROC) curve analysis was employed to evaluate the auxiliary value of serum and aqueous humor IL-20 and Apelin-13 levels in predicting the efficacy of cataract surgery by phacoemulsification combined with IOL implantation. A *p* value < 0.05 was considered statistically significant.

## 3 Results

### 3.1 Comparison of baseline data between the two groups

This study ultimately included 193 cataract patients (Figure 1), with a mean age of (66.98 ± 7.19) years, including 107 males and 86 females. Based on the efficacy evaluation results at 3 months postoperatively, they were divided into a good outcome group (*n* = 138) and a Poor outcome group (*n* = 55). Comparison of clinical baseline data between the two groups showed no significant differences (all *p* > 0.05) in terms of age, gender, BMI, disease duration, operated eye, preoperative BCVA (LogMAR), intraocular pressure, axial length, preoperative CMT, smoking history, alcohol history, history of hypertension, history of hyperlipidemia, phacoemulsification time, phacoemulsification energy, and PCT levels (Supplementary Table S1). While, significant differences (all *p* < 0.05) were observed in diabetes history, CRP levels, and IL-6 levels (Table 1).

**Table 1:** Comparison of baseline data between patients with good and poor postoperative efficacy groups.

Characteristic	All (*n* = 193)	Good efficacy group (*n* = 138)	Poor efficacy group (*n* = 55)	*t*/*χ*^2^	*P*
Age (years)	66.98 ± 7.19	66.32 ± 6.71	68.20 ± 7.63	2.793	0.096
Gender [case (%)]				0.157	0.689
Male	107 (55.44)	75 (54.35)	32 (58.18)		
Female	86 (44.56)	63 (45.65)	23 (41.82)		
BMI (kg/m^2^) disease duration (year)	24.56 ± 3.29	24.28 ± 3.19	25.21 ± 3.53	1.607	0.278
	6.56 ± 1.53	6.21 ± 1.21	7.19 ± 1.79	1.962	0.089
Surgical eye (case, %)				0.248	0.621
Left	98 (50.78)	71 (51.45)	27 (49.09)		
Right	95 (49.22)	67 (48.55)	28 (50.91)		
Preoperative BCVA (LogMAR)	0.54 ± 0.13	0.53 ± 0.10	0.55 ± 0.13	1.069	0.324
Preoperative intraocular pressure (mmHg)	15.49 ± 2.79	15.32 ± 2.39	15.79 ± 2.80	0.972	0.329
Axial length (mm)	23.55 ± 1.32	23.38 ± 1.26	23.79 ± 1.23	0.967	0.398
Preoperative CMT (μm)	214.05 ± 18.90	214.39 ± 18.75	212.83 ± 17.79	1.383	0.176
Drinking history (case, %)	55 (28.50)	40 (28.99)	15 (27.27)	0.126	0.796
Smoking history (case, %)	72 (37.31)	52 (37.68)	20 (36.36)	0.131	0.785
History of hypertension (case, %)	22 (11.40)	15 (10.87)	7 (12.72)	0.329	0.508
History of hyperlipidemia (case, %)	13 (6.74)	9 (6.52)	4 (7.27)	0.230	0.686
History of diabetes (case, %)	11 (5.70)	7 (5.07)	4 (7.27)	1.797	0.039
Phacoemulsification time (s)	18.80 ± 2.79	18.67 ± 2.34	19.08 ± 2.73	0.703	0.679
Phacoemulsification energy (%)	10.85 ± 2.30	10.79 ± 2.19	11.03 ± 2.12	0.683	0.685
CRP (mg/L)	9.89 ± 1.32	8.90 ± 1.26	12.06 ± 1.39	3.201	0.009
IL-6 (pg/mL)	43.25 ± 4.39	39.32 ± 4.58	49.15 ± 4.28	5.893	<0.001
PCT (pg/mL)	17.56 ± 1.90	17.01 ± 1.98	18.14 ± 2.09	2.849	0.079

BMI, body mass index; WBC, white blood cell; CRP, C-reactive protein; IL-6, interleukin-6; PCT, procalcitonin.

The poor outcome group (*n* = 55), the primary reasons for suboptimal results were: cystoid macular edema (CME) in 22 patients (40.0%), clinically significant posterior capsular opacification (PCO) in 15 patients (27.3%), persistent anterior chamber inflammation requiring prolonged treatment in 10 patients (18.2%), and corneal edema in 5 patients (9.1%). Three patients (5.5%) had unexplained suboptimal best-corrected visual acuity (BCVA > 0.3 LogMAR) without a identified complication.

### 3.2 Comparison of serum and aqueous humor IL-20 and Apelin-13 levels between the two groups

This study statistically compared the levels of the biomarkers IL-20 and Apelin-13 between patients in the good outcome group (*n* = 138) and the Poor outcome group (*n* = 55). As shown in Table 2, comparisons of serum and aqueous humor IL-20 and Apelin-13 levels between the two groups showed significant differences. Patients in the good outcome group had significantly lower serum IL-20 levels than those in the Poor outcome group (18.39 ± 4.24 pg./mL vs. 22.79 ± 5.03 pg./mL, *p* < 0.001), while patients in the good outcome group had significantly higher serum Apelin-13 levels than those in the poor outcome group (32.56 ± 6.83 pg./mL vs. 28.39 ± 5.67 pg./mL, *p* < 0.001) (Table 2).

**Table 2:** Comparison of IL-20 and Apelin-13 levels in serum and aqueous humor between two groups of patients.

Item	Good efficacy group (*n* = 138)	Poor efficacy group (*n* = 55)	*t*	*P*
Serum IL-20 (pg/mL)	18.39 ± 4.24	22.79 ± 5.03	5.427	<0.001
Aqueous humor IL-20 (pg/mL)	15.23 ± 3.90	24.63 ± 5.23	11.873	<0.001
Serum Apelin-13 (pg/mL)	32.56 ± 6.83	28.39 ± 5.67	3.572	<0.001
Aqueous humor Apelin-13 (pg/mL)	28.72 ± 5.27	18.90 ± 4.86	10.524	<0.001

IL-20, interleukin-20.

Aqueous humor Apelin-13 and IL-20 levels showed similar trends. Patients in the good outcome group had significantly lower aqueous humor IL-20 levels than those in the Poor outcome group (15.23 ± 3.90 pg./mL vs. 24.63 ± 5.23 pg./mL, *p* < 0.001), while patients in the good outcome group had significantly higher aqueous humor Apelin-13 levels than those in the poor outcome group (28.72 ± 5.27 pg./mL vs. 18.90 ± 4.86 pg./mL, *p* < 0.001). In addition, the intergroup differences were greater in aqueous humor than in serum (Table 2).

### 3.3 Logistic regression analysis of factors influencing postoperative efficacy

**Figure 2:**
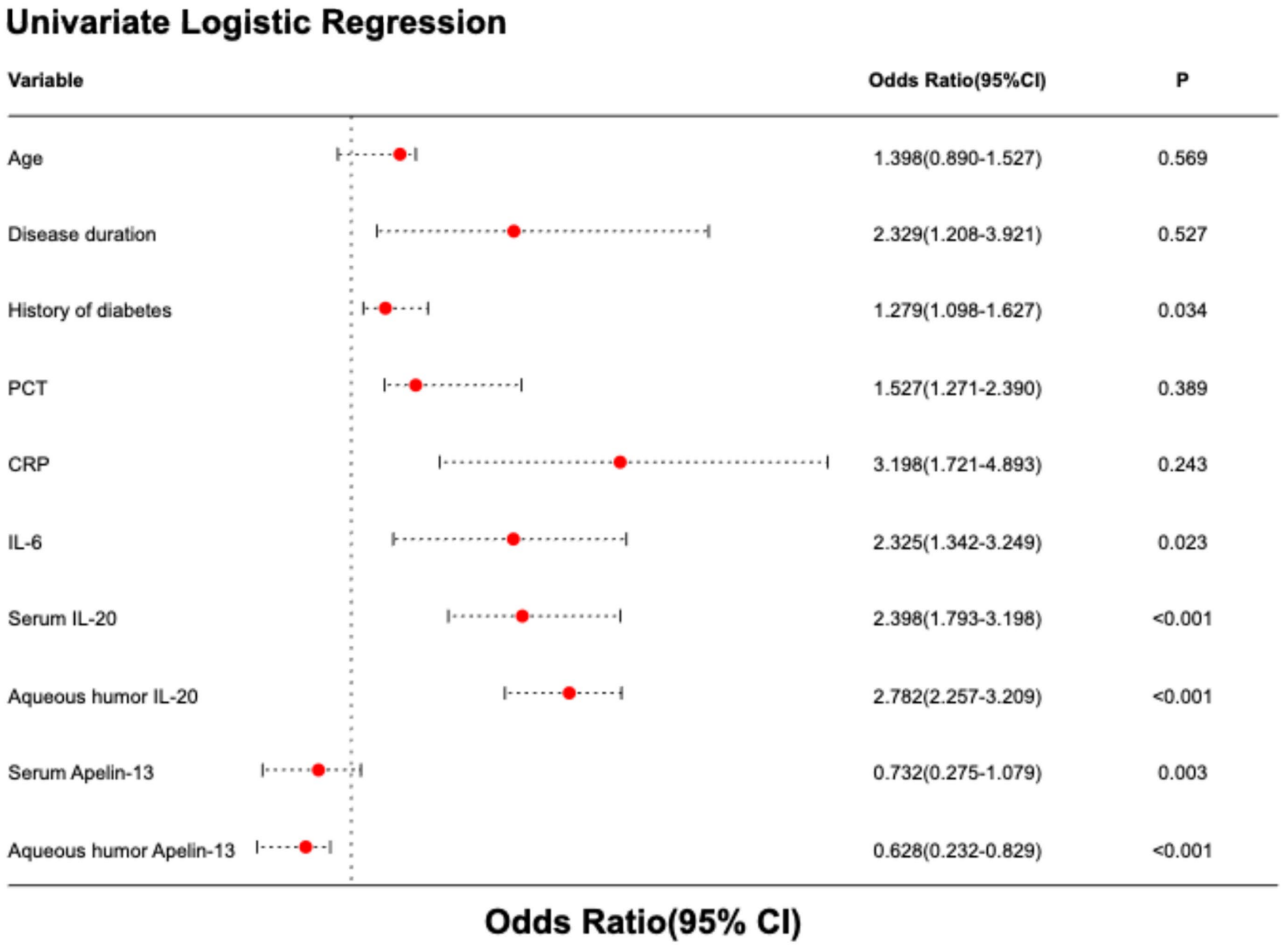
Forest plot of univariate logistic regression analysis of factors influencing postoperative efficacy of cataract surgery.

In this study, the outcome of cataract surgery by phacoemulsification combined with IOL implantation (0 = good outcome, 1 = poor outcome) was set as the dependent variable, and indicators from Table 1 with *p* < 0.1 were included as independent variables in the univariate logistic regression analysis. The results indicated that history of diabetes, IL-6 levels, serum IL-20, aqueous humor IL-20, serum Apelin-13, and aqueous humor Apelin-13 levels were all factors associated with poor postoperative outcome (all *p* < 0.05, Table 3 and Figure 2).

**Table 3:** Univariate and multivariate logistic regression analysis of factors influencing postoperative efficacy of cataract surgery.

Variable	Univariate logistic regression analysis		Multivariate logistic regression analysis	
	*OR* (95% CI)	*P*	*OR* (95% CI)	*P*
Age (years)	1.398 (0.890–1.527)	0.569		
Disease duration (year)	2.329 (1.208–3.921)	0.527		
History of diabetes	1.279 (1.098–1.627)	0.034		
PCT	1.527 (1.271–2.390)	0.389		
CRP	3.198 (1.721–4.893)	0.243		
IL-6	2.325 (1.342–3.249)	0.023	1.893 (1.276–3.192)	0.043
Serum IL-20	2.398 (1.793–3.198)	<0.001	2.179 (1.532–2.798)	0.039
Aqueous humor IL-20	2.782 (2.257–3.209)	<0.001	2.538 (1.679–2.890)	0.027
Serum Apelin-13	0.732 (0.275–1.079)	0.003	0.683 (0.239–0.923)	0.034
Aqueous humor Apelin-13	0.628 (0.232–0.829)	<0.001	0.653 (0.273–0.890)	0.023

OR, odds ratio; 95% CI, 95% confidence interval; CRP, C-reactive protein, IL-6, interleukin-6; IL-20, interleukin-20.

**Figure 3:**
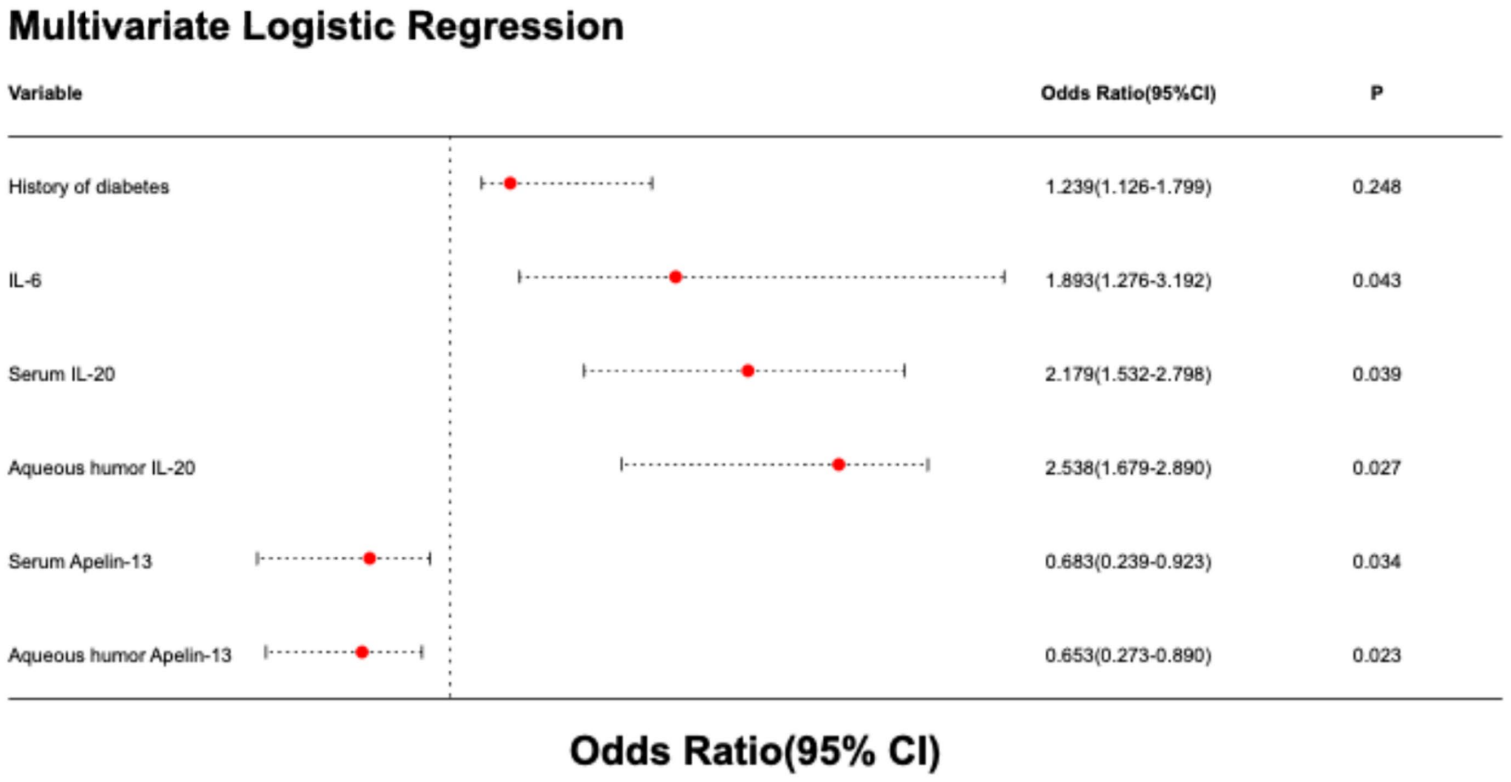
Forest plot of multivariate logistic regression analysis on factors influencing postoperative efficacy of cataract surgery.

Further multivariate logistic regression analysis showed that the OR values for serum IL-20, aqueous humor IL-20, serum Apelin-13, and aqueous humor Apelin-13 levels were 2.179 (95% CI 1.532–2.798), 2.538 (95% CI 1.679–2.890), 0.683 (95% CI 0.239–0.923), and 0.653 (95% CI 0.273–0.890), respectively. High levels of IL-20 and low levels of Apelin-13 in both serum and aqueous humor were independent predictors of poor outcome after cataract surgery by phacoemulsification combined with IOL implantation (all *p* < 0.05, Table 3 and Figure 3).

To further investigate the potential confounding role of diabetes, a sensitivity analysis was performed after excluding all 11 patients with a history of diabetes (final cohort: *n* = 182). In this diabetes-free subpopulation, the associations for aqueous humor biomarkers remained robust. Elevated aqueous humor IL-20 (Adjusted OR: 1.40, 95% CI: 1.19–1.65, *p* < 0.001) and decreased aqueous humor Apelin-13 (Adjusted OR: 0.85, 95% CI: 0.77–0.94, *p* = 0.002) persisted as independent predictors after adjusting for IL-6. Serum IL-20 also retained significance (aOR: 1.15, 95% CI: 1.03–1.29, *p* = 0.012). However, the association for serum Apelin-13 was attenuated (aOR: 0.94, 95% CI: 0.87–1.01, *p* = 0.089). Full results are presented in Table 4.

**Table 4:** Sensitivity analysis: multivariate logistic regression for predictors of poor postoperative outcome in the diabetes-free cohort (*n* = 182).

Variable	A Odds Ratio (aOR) (*n* = 138)	95% Confidence interval	*P*
IL-6 (pg/mL)	1.04	0.99–1.10	0.089
Serum IL-20 (pg/mL)	1.15	1.03–1.29	0.012
Serum Apelin-13 (pg/mL)	0.94	0.87–1.01	0.089
Aqueous Humor IL-20 (pg/mL)	1.40	1.19–1.65	<0.001
Aqueous Humor Apelin-13 (pg/mL)	0.85	0.77–0.94	0.002

The multivariate analysis of the full cohort suggests that the biomarker associations are not merely reflective of diabetic status, as diabetes itself was not an independent predictor when biomarkers were considered. This conclusion is strengthened by the sensitivity analysis, which demonstrates that the strong associations for aqueous humor IL-20 and Apelin-13 persist in a cohort entirely free of diabetes.

### 3.4 ROC curve analysis of the predictive value of IL-20 and Apelin-13 for poor postoperative outcome

This study used ROC curve analysis to evaluate the predictive value of serum and aqueous humor IL-20 and Apelin-13 levels for poor postoperative outcomes in patients undergoing cataract surgery by phacoemulsification combined with IOL implantation.

**Figure 4:**
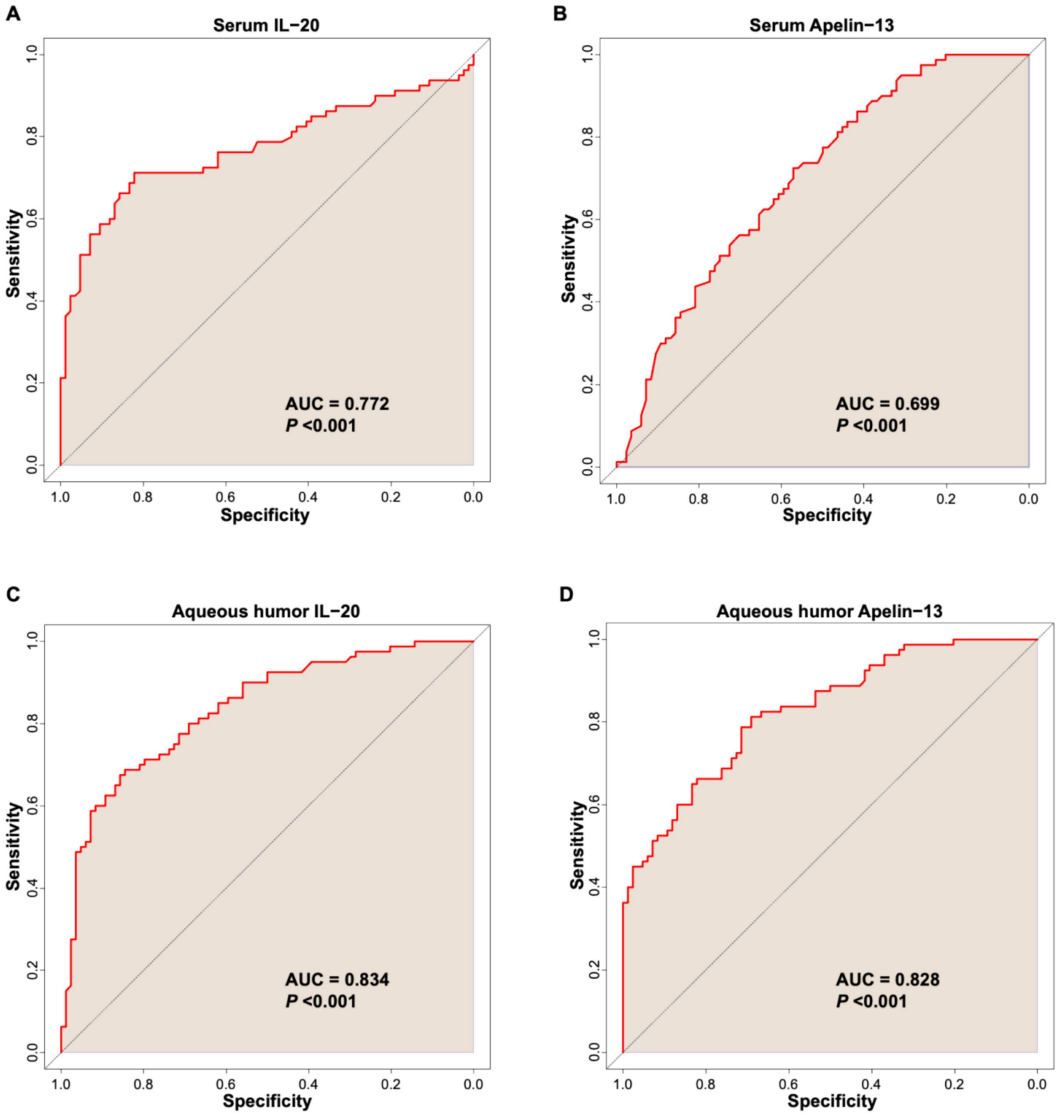
ROC curve of serum IL-20 **(A)**, serum Apelin-13 **(B)**, aqueous humor IL-20 **(C)**, and aqueous humor Apelin-13 **(D)** levels for predicting poor efficacy of phacoemulsification combined with IOL implantation surgery in cataract patients.

As shown in Figure 4, ROC curve analysis revealed that the AUC for serum IL-20 in predicting poor postoperative outcome was 0.772 (95% CI: 0.697–0.848), with a sensitivity of 71.30% and specificity of 76.10% (Figure 4A and Table 5). The AUC for serum Apelin-13 in predicting poor postoperative outcome was 0.699 (95% CI: 0.619–0.778), with a sensitivity of 72.50% and specificity of 73.60% (Figure 4B and Table 5). The AUC for aqueous humor IL-20 in predicting poor postoperative outcome was 0.834 (95% CI: 0.773–0.896), with a sensitivity of 77.80% and specificity of 84.50% (Figure 4C and Table 5). The AUC for aqueous humor Apelin-13 in predicting poor postoperative outcome was 0.828 (95% CI: 0.767–0.889), with a sensitivity of 78.90% and specificity of 82.50% (Figure 4D and Table 5). These results indicate that aqueous humor IL-20 and Apelin-13 levels have good predictive ability for the outcome of cataract surgery by phacoemulsification combined with IOL implantation. Levels of IL-20 and Apelin-13 in aqueous humor are useful for predicting postoperative outcome.

**Table 5:** ROC curve analysis results of serum and aqueous humor IL-20, Apelin-13, and IL-20/Apelin-13 ratio for predicting poor postoperative efficacy.

Item	AUC	95% CI	Sensitivity	Specificity	*P*	Cutoff value
Serum IL-20 (pg/mL)	0.772	0.697–0.848	71.30%	76.10%	<0.001	≥20.56
Aqueous humor IL-20 (pg/mL)	0.834	0.773–0.896	77.80%	84.50%	<0.001	≥18.90
Serum Apelin-13 (pg/mL)	0.699	0.619–0.778	72.50%	73.60%	<0.001	≤28.79
Aqueous humor Apelin-13 (pg/mL)	0.828	0.767–0.889	78.90%	82.50%	<0.001	≤22.48
Serum IL-20/Apelin-13 ratio	0.795	0.727–0.862	73.70%	85.30%	<0.001	≥0.76
Aqueous humor IL-20/Apelin-13 ratio	0.856	0.803–0.912	80.90%	83.50%	<0.001	≥0.79

AUC, area under curve; 95% CI, 95% confidence interval; IL-20, interleukin-20.

**Figure 5:**
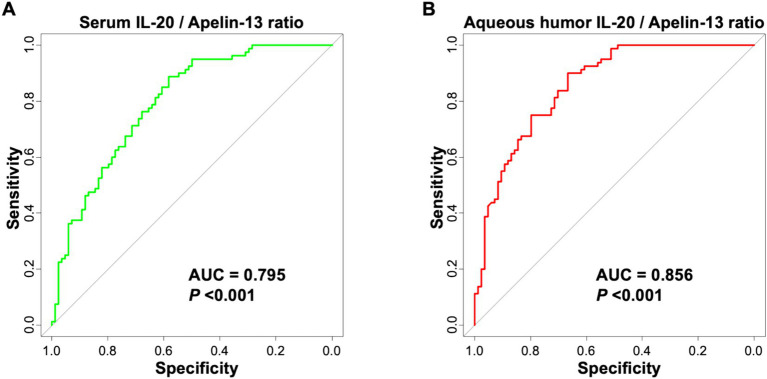
ROC curve of serum IL-20/Apelin-13 ratio **(A)** and aqueous humor IL-20/Apelin-13 ratio **(B)** for predicting poor efficacy of phacoemulsification combined with IOL implantation surgery in cataract patients.

Combining IL-20 and Apelin-13, the serum IL-20/Apelin-13 ratio achieved an AUC of 0.795 (95% CI: 0.727–0.862) for predicting poor postoperative outcome, with a sensitivity of 73.70% and specificity of 85.30% (Figure 5A and Table 5). The aqueous humor IL-20/Apelin-13 ratio achieved an AUC of 0.856 (95% CI: 0.803–0.912) for predicting poor postoperative outcome, with a sensitivity of 80.90% and specificity of 83.50% (Figure 5B and Table 5). These results indicate that the aqueous humor IL-20/Apelin-13 ratio has good predictive ability for the outcome of cataract surgery by phacoemulsification combined with IOL implantation.

## 4 Discussion

Phacoemulsification combined with IOL implantation is a safe and effective sight-restoring surgery, yet a proportion of patients experience suboptimal recovery. Identifying preoperative biomarkers associated with this risk could enable personalized management. This retrospective study is the first to explore the relationship between IL-20 and Apelin-13 levels and a composite postoperative outcome following cataract surgery. Our principal findings indicate that elevated IL-20 and reduced Apelin-13 levels in both serum and aqueous humor are associated with an increased likelihood of a suboptimal postoperative course, defined as BCVA > 0.3 LogMAR and/or the occurrence of complications.

A critical consideration in interpreting our findings is the use of a composite endpoint. While this pragmatic definition captures a broad spectrum of suboptimal recovery, it groups patients with different pathophysiologies, which may dilute specific biomarker-complication relationships. Furthermore, the significant univariate association of diabetes and elevated systemic inflammatory markers (IL-6, CRP) with poor outcome raised the possibility that IL-20 and Apelin-13 might simply reflect a diabetes-driven pro-inflammatory state rather than surgery-specific pathology. However, our multivariate analysis, adjusted for IL-6, demonstrated that the biomarker associations remained significant, while the history of diabetes did not retain independent predictive value. More conclusively, a sensitivity analysis excluding all diabetic patients confirmed that elevated aqueous humor IL-20 and reduced aqueous humor Apelin-13 were robustly associated with poor outcomes in a diabetes-free cohort. These analyses suggest that while systemic inflammation is a powerful co-factor, IL-20 and Apelin-13 in the ocular microenvironment provide additional, specific prognostic information. They may indicate a pre-existing state of altered ocular immune homeostasis or tissue resilience that predisposes to a more pronounced postoperative response.

Apelin-13, as an active fragment of the Apelin/APJ system, is widely expressed in tissues and possesses multiple effects, including anti-inflammatory, antioxidant, promotion of vascular homeostasis, and neuroprotection (17, 21–23). This study is the first to report the relationship between Apelin-13 and the outcome of cataract surgery by phacoemulsification combined with IOL implantation, finding that high expression levels of Apelin-13 in serum and aqueous humor are protective factors for good postoperative visual function recovery.

The protective mechanisms of Apelin-13 may involve several aspects. Firstly, Apelin-13 may regulate the inflammatory response that occurs after cataract surgery. After phacoemulsification for cataracts, there will be a certain degree of inflammatory response in the eye, manifested as an increase in cytokine levels in the aqueous humor (24, 25). For example, elevated levels of uric acid in the aqueous humor of cataract patients can stimulate aging of lens epithelial cells (LECs), leading to cataract progression, which is associated with activation of NLRP3 inflammasomes (26). A previous study showed that Apelin-13 can inhibit inflammatory responses by activating the PI3K/Akt/eNOS signaling pathway, thereby alleviating surgical trauma-induced damage to corneal endothelial cells and retinal tissues (18). Apelin-13 can affect the proliferation, migration, and angiogenesis of endothelial cells, suggesting its possible involvement in vascular repair and inflammation resolution processes (27). In addition, cytokine levels such as IFN-*γ*, TNF-*α*, and IL-1β in aqueous humor were significantly elevated in patients with uveitis, while irisin levels were decreased and negatively correlated with TNF-α (28). Apelin-13, as a peptide with anti-inflammatory potential, its high expression may help inhibit postoperative inflammatory response and promote the recovery of eye tissue. The biocompatibility of artificial intraocular lenses is crucial for visual reconstruction after cataract surgery (29). The common complication after IOL implantation is posterior capsule opacity (PCO) (30, 31). PCO is associated with the proliferation, migration, and inflammatory response of lens epithelial cells. High levels of Apelin-13 may reduce the incidence of PCO, optimize the biocompatibility of IOL, and improve postoperative efficacy by inhibiting postoperative inflammation. Secondly, Apelin-13 has antioxidant effects, reducing the accumulation of reactive oxygen species and subsequent damage to corneal endothelial cells and retinal pigment epithelial cells (32). Apelin-13 can also regulate glucose metabolism, promote energy supply, and enhance tissue repair capacity. Previous research indicated that Apelin-13 can counteract optic nerve injury in glaucoma by modulating glucose metabolism (17). Similar mechanisms might be involved in postoperative repair after cataract surgery.

Both aqueous humor and serum are potential sources of biomarkers for disease diagnosis and prognostic evaluation (20, 33). A study has conducted a correlation analysis between electrolytes in aqueous humor and serum, indicating a certain correlation between these two body fluids (33). In addition, there are differences in cytokine levels between aqueous humor and plasma in patients with uveitis, with certain cytokines having significantly higher concentrations in aqueous humor than in plasma (34). Lower levels of Apelin-13 in serum and aqueous humor may indicate insufficient anti-inflammatory and vascular protective mechanisms in the patient’s body, making them more susceptible to complications such as macular edema postoperatively, thereby affecting visual recovery and efficacy after cataract surgery.

IL-20 is a member of the IL-10 cytokine family, primarily involved in inflammatory responses and angiogenesis processes (10). In ophthalmology, IL-20 has been found to be elevated in some ocular inflammatory diseases. Yang et al. reported a positive correlation between serum IL-20 levels and the occurrence of dry eye syndrome after cataract surgery (*r* = 0.45, *p* = 0.015) (11), potentially mediated by IL-20 promoting inflammatory cell infiltration and inhibiting lacrimal gland function via IL-20 receptor activation of the STAT3 signaling pathway, leading to the release of various inflammatory factors. Furthermore, IL-20 might influence corneal wound healing by promoting the expression of matrix metalloproteinases.

The stronger associations and higher predictive values observed for aqueous humor biomarkers compared to serum are biologically plausible. Aqueous humor directly bathes the intraocular tissues involved in surgical trauma and healing, making it a more direct reflection of the local pathophysiological state than systemic circulation (20). The blood-aqueous barrier likely contributes to this compartmentalization (34). Further explored the ratio of IL-20 to Apelin-13 as a single metric representing the balance between pro-inflammatory and protective signals. The finding that this ratio (particularly in aqueous humor) yielded the highest AUC (0.856) is a novel and exploratory result. It suggests that the functional equilibrium between these counteracting pathways may be more informative than either marker alone. However, we emphasize that this ratio and its optimal cutoff were derived from this specific cohort and require external validation.

In the eye, aqueous humor is the transparent fluid filling the anterior and posterior chambers, and its composition and circulation are crucial for maintaining intraocular environmental homeostasis (20). Serum is a source of biomarkers in the systemic circulation, reflecting systemic inflammatory and immune status (35). This study found that the intergroup differences and predictive value of IL-20 and Apelin-13 levels were greater in aqueous humor than in serum, indicating that these two factors primarily act locally within the eye, and their aqueous humor levels better reflect intraocular pathological states. This could be due to the blood-aqueous barrier, causing discrepancies between serum and aqueous humor factor levels. Therefore, for biomarker research in ocular diseases, direct detection of aqueous humor might be more clinically significant than serum.

The AUC values for aqueous humor IL-20 (0.834), Apelin-13 (0.828), and their ratio (0.856) indicate good discriminative ability within our study population. It is crucial, however, to interpret these metrics with caution in the clinical context. These values, derived from a retrospective, single-center analysis, are subject to optimism bias. The associated sensitivity and specificity estimates mean that a clinically relevant proportion of patients would be misclassified. Therefore, these biomarkers are not presented as standalone diagnostic tests but as promising candidates for inclusion in future multivariable risk prediction models.

Our study has important limitations that must be acknowledged. First, the retrospective design and composite outcome definition introduce potential bias and limit the specificity of our conclusions. Second, the 3-month follow-up period is adequate for assessing early recovery but is too short to evaluate late-onset complications like significant posterior capsular opacification. Third, while we addressed diabetes through sensitivity analysis, residual confounding from other unmeasured systemic or ocular factors cannot be excluded. Fourth, the sample size, while adequate for initial biomarker discovery, limited the complexity of our multivariate models and necessitates validation in larger cohorts. Finally, our measurements relied on ELISA without orthogonal validation, and the mechanistic roles of IL-20 and Apelin-13 in the context of cataract surgery recovery remain speculative, warranting confirmation in experimental models.

## 5 Conclusion

This study is the first to confirm that IL-20 and Apelin-13 levels are closely associated with the efficacy of phacoemulsification combined with intraocular lens (IOL) implantation for cataract treatment. Apelin-13 serves as a protective factor for favorable postoperative outcomes, while IL-20 acts as a risk factor. The levels of IL-20 and Apelin-13 in the aqueous humor, as well as the IL-20/Apelin-13 ratio, exhibit strong predictive value for poor postoperative efficacy, with the IL-20/Apelin-13 ratio demonstrating the highest predictive value.

Taken together, these findings provide novel biomarkers for preoperative risk assessment of cataract surgery, offering a theoretical basis for personalized risk evaluation and targeted treatment strategy formulation, with significant clinical implications for improving postoperative outcomes. Future research with larger sample sizes is needed to further validate the clinical application value of IL-20 and Apelin-13.

## Data Availability

The original contributions presented in the study are included in the article/Supplementary material, further inquiries can be directed to the corresponding author/s.
