# Supplementary material for: Efficacy evaluation and predictive value of IL-20 and Apelin-13 after cataract surgery by phacoemulsification combined with IOL implantation
Source: Front Med (Lausanne). 2026 Jan 13;12:1737194. doi: 10.3389/fmed.2025.1737194 (PMC12835301; doi:10.3389/fmed.2025.1737194)
Supplement: Supplementary file 1 [file Table_1.DOCX]

**Supplementary Table S1:** Patients in the Poor Outcome Group (n=55)

| Category | Subgroup | n | BCVA Status (LogMAR) | Complication Type |
| --- | --- | --- | --- | --- |
| Vision Loss Only | A | 03 | > 0.3 | None |
| Good Vision with Complications | B | 27 | ≤ 0.3 | Yes (Total) |
|  | B1 | 05 | ≤ 0.3 | Transient Corneal Edema |
|  | B2 | 05 | ≤ 0.3 | Persistent Inflammation |
|  | B3 | 10 | ≤ 0.3 | Cystoid Macular Edema (CME) |
|  | B4 | 07 | ≤ 0.3 | Posterior Capsular Opacification (PCO) |
| Vision Loss AND Complications | C | 25 | > 0.3 | Yes (Total) |
|  | C1 | 12 | > 0.3 | With CME |
|  | C2 | 08 | > 0.3 | With PCO |
|  | C3 | 05 | > 0.3 | With Persistent Inflammation |
| Unexplained | U* | 03 | > 0.3 | None |
| TOTAL |  | 55 |  |  |
